# Staphyloraphy and Uranoplasty

**Published:** 1874-02

**Authors:** John E. Owens

**Affiliations:** Chicago


A MONTHLY RECORD OF

*Medicine, Surgery and the Collateral Sciences.*

EDITED BY J. ADAMS ALLEN, M.D., LL.D.; AND WALTER HAY, M.D.

VOL. XXXI. — FEBRUARY, 1874. — NO. 2.

### Original Communications.

ARTICLE I.—*Staphyloraphy and Uranoplasty.* By JNO. E. OWENS, M.D. Read before the Chicago Society of Physicians and Surgeons, August 26th, 1873.

We have no record of Staphyloraphy, much less of Uranoplasty, amongst the ancients. The honor of the first successful case of the former is generally conceded to Roux, of Paris, but the operation dates somewhat further back than its performance by this surgeon. Robert, in his "Memoirs on Various Medical Subjects," published in 1764, says: "A child had the palate cleft from the velum to the incisor teeth. Lemonnier, a very skillful dentist, endeavored and succeeded in reuniting the two borders of the cleft, first inserted several points of suture in order to keep them approximated, and afterwards abraded them with a cutting instrument. An inflammation supervened, which terminated in suppuration, and was followed by the reunion of the two lips of the artificial wound. The child was perfectly cured." After making every allowance for the inaccuracy of the times, one can scarcely doubt that Lemonnier's patient had a cleft palate, and that Lemonnier himself performed a staphyloraphy, and perhaps, also, uranoplasty. Forty-nine years later (1813), M. Colombe made various trials of the operation upon the dead subject, and two years afterwards, a patient upon whom he wished to operate, refused. It seems, then, that the operation is entirely French. Graefe, in 1817, published the details of its performance (staphyloraphy), dating his operation at the end of 1816. His effort was unsuccessful, and barely escaped notice. In 1819, Stephenson, a young physician, presented to Roux the first opportunity for the performance of staphyloraphy, which proved successful, and Stephenson himself published his cure in a thesis in 1821, in London. One year later, Mr. Alcock, probably enlightened by Stephenson's paper, was as successful as his French cotemporary. It was at this time that a controversy arose as to the right of Graefe or Roux to priority. The latter, it seems, was not aware of what had been done by his neighbor across the Rhine. We have strong reason, however, to believe, as already mentioned, that its performance antedates both that of Roux and Graefe. Shortly after Roux's operation, Dr. Jno. C. Warren, of Boston, not being aware of what had been done in Europe, performed the same, and invented new instruments for it. Under favorable circumstances, staphyloraphy is neither very difficult nor very painful, but it is a long and delicate operation, and requires much endurance on the part of the patient and surgeon. The former must be sensible of the importance of the operation, and not only willing, but capable of cooperating with the surgeon, otherwise the task becomes one of the most difficult in surgery—often, indeed, an impossibility. To attempt a detailed description of the operation as performed by different surgeons, or the instruments used by them, would tax the patience of this audience too severely, and perhaps unnecessarily lengthen this paper. Suffice it to say, that the operation consists of three steps, viz.: paring the edges of the cleft; introducing the sutures; approximating the flaps and knotting the threads.

In 1843, staphyloraphy was probably seventy-nine years old. It had been performed many times in this country, and in France, Germany, and England, with many successes and many failures. In this year (1843), Dr. J. Mason Warren, of Boston, published in the *New England Quarterly Journal of Medicine and Surgery* an account of an important modification of the operation of staphyloraphy. This modification by Dr. Warren renewed the interest of the profession, and divulged the secret of many failures. Here are Dr. Warren's own words: "The improvement to which I alluded, in the operation upon the soft palate, consisted in the relaxation of the tissues of the fissured velum, by means of incisions, made with strong curved scissors, so as to divide the attachments of the soft palate to the tonsils, and to the posterior pillar, or, in other words, dividing the posterior pillar of the palate just where it begins to spread out into the velum. The effect of this incision is at once seen in the almost complete relaxation of the parts." Again, the same author says: "In subsequent operations, however, I found that there existed, in some cases, an additional obstacle to the approximation of the flaps, which could be overcome as easily, and in the same manner as the former. This obstacle consists of a band of firm tissue, extending above and behind the soft palate, and standing in bold relief when that organ is put on the stretch by drawing upon it with the forceps. This resisting mass, like the other, I have always divided by an additional stroke or two with the scissors, whenever the incision of the posterior pillar and adjacent mucous membrane has seemed insufficient properly to relax the palate."

One year later, 1844, Mr. Fergusson, now Sir Wm. Fergusson, after the dissection of a specimen of cleft palate, adopted a method of relief to the tension of the flaps, very similar to that of Warren's, last described. The muscles divided by both Warren and Fergusson are the levator palati, the palato-pharyngeus, and sometimes the palato-glossus on each side. The levator palati muscle is best reached by Pollock's method, in which a narrow, sharp-pointed knife is passed upwards and backwards, and somewhat inwards, through the palate, close to, a little in front and to the main side of the hamular process of the sphenoid bone. (Location of process.) By this relief of tension staphyloraphy seems to have been perfected in its essential features, and the percentage of failures greatly reduced.

Now, gentlemen, all that has been said as yet in this paper refers to the operation as applied to adults, and without the aid of anæsthetics. In nearly all of our surgical treatises we are directed to wait till the period of puberty, at least, before attempting the cure of such malformations as those under discussion, the patient not being capable before that time of rendering to the surgeon the required assistance. Of late, however, it has been shown conclusively that the operation can be successfully performed on very young children, infants indeed, and that, too, with the aid of chloroform. Mr. Thos. Smith, of St. Bartholomew's Hospital, was amongst the first to introduce chloroform in operations on the palate in children. The same surgeon has further facilitated the various steps of the operation by the application of a gag for keeping the jaws apart during the operation. His paper was dated Jan. 14, 1868. From the "Surgical Treatment of Children's Diseases, by Holmes," 1869, I extract this note, which shows that Mr. Smith was not the first to use chloroform in this operation. In the *Dublin Quarterly Jounal* for Nov. 1, 1867, the following sentence occurs: "Mr. Collis, of the Meath Hospital, gives it (chloroform) habitually, and has been able to operate with success on very young children." "Mr. Durham, also, had operated four times under chloroform before the date of Mr. Smith's paper." "In one case aged (8½ yrs.) complete success was obtained in two operations." "The other two cases did not succeed." A successful case at the age of four months, operated on by Mr. F. Buszard, of Northampton, Dec. 15, 1867, will be found reported in the *Brit. Med. Jour.*, April 11, 1868.

The following table of cases gleaned from the last mentioned work, exhibits, so far as they go, the results of these operations, probably on the soft palate, under the influence of chloroform. I have added my own (on the hard palate) under the influence of æther:

| No. | Age. | Result. | Surgeon. | Degree. | No. opera's. | Remarks. |
|---|---|---|---|---|---|---|
| 1 | 6 m. | Failure. | Billroth. | 0 | 0 | |
| 1 | 0 | " | " | " | " | |
| 1 | 28 wks. | Successful. | " | Complete. | 3 | Langenbeck's method. |
| 1 | 0 | Failure. | " | 0 | 0 | Some union. |
| 1 | 0 | " | " | 0 | 0 | |
| 1 | Infancy. | " | Langenbeck. | 0 | 0 | |
| 1 | " | " | " | 0 | 0 | |
| 1 | 2 yrs. | " | Smith. | 0 | 2 | 1 failed from scarlatina. 1 union had taken place, but cough set in and a good deal gave way. |
| 1 | 2 yrs 11 m. | Doubtful. | " | 0 | 2 | |
| 1 | 3 yrs. 2 m. | Probably successful. | " | " | 2 | Last seen a week after operation—had every appearance of success. |
| 1 | 5 yrs. | Successful. | " | 0 | 0 | |
| 1 | 6 yrs. | " | " | 0 | 0 | |
| 1 | 9 yrs. | " | " | 0 | 0 | |
| 1 | 10 yrs. | " | " | 0 | 0 | |
| 1 | 13 yrs. | " | " | 0 | 0 | |
| 1 | 13 yrs. | | " | 0 | 0 | Operation had only just been performed. |
| 1 | 8½ yrs. | Successful. | Durham. | 0 | 2 | |
| 1 | Childhood. | Failure. | " | 0 | 0 | |
| 1 | " | " | " | 0 | 0 | |
| 1 | 4 m. | Successful. | Buszard. | 0 | 0 | |
| 1 | 11 | " | Owens. | Complete. | 1 | Operation upon hard palate only. Completed with æther. |

Of the twenty-one cases tabulated, nine were successful, one was doubtful, one probably successful, having been last seen a week after the operation with every prospect of success; in one the operation had just been performed, and nine were failures. Mr. Smith, with the aid of his gag and chloroform, nine cases. Five of these were successful, one doubtful (scarlatina), one probably successful; in one the operation had just been performed, and one operation failed. The ages ranged from four months to thirteen years. Although Mr. Smith cannot claim precedence in the performance of this operation on young children, with the aid of chloroform, it is certain that his name is well known in connection with it, and that he has done more than any other surgeon to bring it to the notice of the profession. He has likewise obtained the most gratifying results. Indeed, gentlemen, the operation of staphyloraphy has been now so perfected at home and abroad, that there is little to look for, perhaps, beyond improvement in operative details. We may the better appreciate this, when we reflect upon the fact that the earlier attempts at abrasion of the cleft were made with the mineral acids, caustic potash, tr. canthæ, lapis infernalis, and the hot iron.

We will now leave the subject of staphyloraphy, or the operation for the closure of clefts in the soft palate, in order to consider uranoplasty, or that operation which has for its object the closure of clefts in the hard palate or roof of the mouth.

In former times, nearly all fissures extending into the hard palate were rejected as unfit for operation. We should not overlook the fact, already mentioned, that the child upon whom Lemonnier operated, as reported by Robert, in 1764, was *perfectly cured*, and that the cleft extended from the velum to the incisor teeth. This is probably not only the earliest record of staphyloraphy, but also the earliest mention of uranoplasty. However, in 1843, when Dr. J. Mason Warren published in the *New England Quarterly Journal of Medicine and Surgery*, the account of his important modification of the operation of staphyloraphy, he included an account of a new operation for the closure of fissures in the hard palate. The right of priority is usually, but it seems erroneously, conceded to Dr. J. Mason Warren. There seems to be some diversity of opinion as to what really is Dr. Warren's operation. In the first volume of Velpeau's Operative Surgery, we find Warren's operation mentioned in the following terms: The soft palate having been prepared for staphyloraphy by the usual abrasion of its edges, and the introduction of the necessary points of suture, the mucous membrane covering the roof of the mouth was carefully raised on each side of the fissure in the hard palate, and when thus detached, they were brought across the fissure and united, like the soft palate, by the interrupted sutures, the flaps formed by the mucous membrane of the mouth being continuous with the denuded edges of the soft palate. This difficult and delicate operation was completely successful, and is by no means diminished in value from having been first devised and successfully performed, many years before, by a German surgeon, named Krimer or Kreimer. Now let us glance at the process of Krimer: This surgeon made incisions at some distance outside a cleft which remained in the hard palate after a staphyloraphy. He was enabled to dissect from the sides towards the cleft two flaps, which he reversed upon themselves, and then united by stitches. The patient made a perfect recovery.

It is plain that Kreimer and Warren performed uranoplasty by different processes, but the right of priority belongs to Kreimer. Gross seems to imply, in the last edition of his System of Surgery, that Warren performed the operation by dissecting the flaps from the alveolar border towards the cleft on each side, whereas he dissected in the opposite direction. In order to get the truth of the matter we naturally consult Warren himself. "The operation," he says, "upon the hard palate, consisted in dissecting up with a long, double-edged knife, curved on its flat side, the membranes covering the hard palate, pursuing the dissection quite back to the root of the alveolar processes." Dr. Warren distinctly states in the same work, "Surgical Observations," that the flaps were made to cover the cleft par glissement. Such are the peculiarities of this infirmity, that it is not by any single operative process that the most gratifying results are obtained. Each case must be studied *per se*, its complications well considered, and the operation modified accordingly. Dr. Wm. R. Whitehead, of N. Y., in 1869, read before the American Medical Association, "A Report on the Best Methods of Treatment for Different Forms of Cleft Palate." This paper contains a description of a very efficient instrument known as Whitehead's gag, the principle of action being the same as that of Smith's. The former is less expensive. Dr. Whitehead etherizes his patients. The use of the gag, the simplification of other instruments, and the use of anæsthetics, have greatly facilitated the performance of staphyloraphy and uranoplasty, made them practicable in the case of children, and have given the operative treatment of cleft palate a high rank in surgery. In all such cases, the infant is unable to suck the nipple, and hence, during the first months, it is a struggle for life. Such infants should be hand-fed, in the upright position. Uranoplasty should not be inflicted upon very young infants, for several reasons, viz.: Infants bear hemorrhage badly, convulsions are likely to supervene; the traumatic inflammation interferes with the feeding of the child. Children do not begin to pronounce many words before the age of two years. The fissure in the soft palate should be first closed, and the next step should be the closure of the hare-lip, should that defect exist, or the reverse. Should these operations be successful, we have every reason to look for a diminution in the cleft of the hard palate in the course of several months. Now, gentlemen, it is the opinion of many of our most eminent surgeons, that uranoplasty in adults, and staphyloraphy too, for that matter, are productive of no very decided improvement in articulation, but if the cleft be closed about the time that articulation commences, or soon after, our patients would doubtless receive a benefit which can scarcely be exaggerated. This was once an operation nearly hopeless in many cases. To-day it can be looked upon as certain to effect the closure of the most extensive cleft, and I hope I shall not be considered too enthusiastic in saying that it is likewise almost sure to improve a most unfortunate articulation. In August, 1867, a little girl aged eight years came under my care, and was admitted to St. Luke's Hospital. She had a cleft extending through the velum, the hard palate, the alveolar process between the incisor teeth, and through the lip. Soon after admission the usual operation for hare-lip was performed, with good result. One of the incisor teeth, in consequence of the deformity, presented one of its sides towards the lip, and so constantly impinged against the line of incision, that ulcerations supervened. Dr. Allport disposed of this inconvenience by turning the tooth in its socket, in order to place the former in normal relation to the lip.

In February, 1871, when the child was nearly twelve years old, and four years after the operation on the lip, I was induced to attempt the closure in the cleft in the hard palate by the following method: An incision, commencing at the posterior margin of the hard palate, was carried forward, close to the alveolar processes, and terminated close to the anterior extremity of the cleft behind the incisor teeth. A second incision was made transversely, connecting the posterior extremity of the cleft with the point of commencement of the incision already described. The flap thus mapped out was dissected up, as close to the bone as possible, from the alveolar processes towards the cleft. A similar flap having been made in the opposite side of the cleft, the two hung vertically, as two curtains, one from each margin of the cleft. A thread of surgeon's silk was then armed at each end with Sim's needle. The flaps hanging as just described, one needle was passed through the posterior end of the flap from without inward, drawn forward as far as was deemed necessary, and made to transfix the flap near its anterior end from within outwards. The other end of the thread was carried in a similar manner through the opposite flap. The flaps were then rolled or crowded up into the fissure, with their raw surfaces in apposition, and the sutures tightened and knotted. By this method the fissure in the hard palate was closed, with the exception of an unimportant orifice just behind the incisor teeth. This was done in the presence of Prof. Gunn and Dr. Allport, and I should not omit to mention that it was the second attempt to close this fissure. The first one I was obliged to abandon at a very early stage, for want of the co-operation of the patient. The second operation was pursued as far as possible without an anæsthetic, and when the patient ceased to co-operate, æther was administered, under the influence of which the operation was completed. The jaws were kept asunder by means of corks. Hemorrhage from the posterior palatine artery continuing after the operation had been completed, was arrested by digital compression. A solution of chlorate of potash was employed, as a mouth wash during the healing process. The advantages claimed for this method of closing wide fissures in the hard palate are the following:

1. There is no tension of the flaps.
2. Two large raw surfaces are placed in contact instead of two raw edges, thereby increasing the chances of success.
3. The dissection from without inward is less difficult than a dissection in an opposite direction.
4. The difficulty of introducing and knotting the suture is reduced to a minimum.
5. Fissures of greater width can be closed by this than by any other method hitherto attempted.

Finally, from a consideration of this subject we have deduced the following conclusions:

1. That the operation for cleft of the hard and soft palate, unless for the purpose of facilitating the nourishment of the child, should not be attempted before the age of two years.
2. That we should not attempt the closure of the cleft in the hard and soft palate at one operation.
3. That the use of an anæsthetic, or the full co-operation of the patient, is necessary to the success of the operation.
4. That the operations in question upon young children, with the aid of an anæsthetic and gag, can be successfully performed, and that we have reason to look for a marked improvement in articulation.
5. That staphyloraphy was performed as early as 1764 by

Lemonnier, and perhaps, also, uranoplasty—48 years before Graefe and 55 years before Roux of Paris performed the former.

6. That both Lemonnier and Krimer performed uranoplasty before it was undertaken by Dr. Jno. Mason Warren.

7. That Dr. Jno. Mason Warren, in 1843, devised the operation for the relaxation of the flaps in staphyloraphy, and that priority belongs to him and not to Fergusson.

